# Effects of Na_2_CO_3_/Na_2_SiO_3_ Ratio and Curing Temperature on the Structure Formation of Alkali-Activated High-Carbon Biomass Fly Ash Pastes

**DOI:** 10.3390/ma15238354

**Published:** 2022-11-24

**Authors:** Chengjie Zhu, Ina Pundienė, Jolanta Pranckevičienė, Modestas Kligys

**Affiliations:** Laboratory of Concrete Technology, Institute of Building Materials, Vilnius Gediminas Technical University, Linkmenų Str. 28, LT-08217 Vilnius, Lithuania

**Keywords:** biomass fly ash, alkali-activated materials, Na_2_CO_3_/Na_2_SiO_3_ ratio, curing temperature, structure formation, compressive strength

## Abstract

This study explored unprocessed high-carbon biomass fly ash (BFA) in alkali-activated materials (AAM) with less alkaline Na_2_CO_3_ as the activator. In this paper, the effects of the Na_2_CO_3_/Na_2_SiO_3_ (C/S) ratio and curing temperature (40 °C and 20 °C) on the setting time, structure formation, product synthesis, and physical-mechanical properties of alkali-activated BFA pastes were systematically investigated. Regardless of curing temperature, increasing the C/S ratio increased the density and compressive strength of the sample while a decrease in water absorption. The higher the curing temperature, the faster the structure evolution during the BFA-based alkaline activation synthesis process and the higher the sample’s compressive strength. According to XRD and TG/DTA analyses, the synthesis of gaylussite and C-S-H were observed in the sample with an increasing C/S ratio. The formation of the mentioned minerals contributes to the compressive strength growth of alkali-activated BFA pastes with higher C/S ratios. The findings of this study contribute to the applicability of difficult-to-recycle waste materials such as BFA and the development of sustainable BFA-based AAM.

## 1. Introduction

Pressures to protect the global environment and provide renewable energy sources have resulted in rising demand for biomass renewable energy sources. Biomass plays a significant role in increasing ash generation, which should be regulated environmentally [1,2]. Biomass ashes, high in carbon due to their biogenic nature, have been one of the most heavily utilized wastes [3]. Primary solid biofuels (plant matter used directly as fuel or processed into solid fuels) represent about 9% of worldwide energy production [4,5]. It is predicted that industrial biomass incineration for combined heat and power will triple by 2035 compared with 2008 levels [6]. This expansion is partly driven by rules that promote biomass as a renewable energy source and burning as a CO_2_-neutral process. In most cases, the inorganic elements of biomass do not burn during the incineration process [7,8,9]. They are left as ashes together with a proportion of unburned carbon, depending on the temperature and efficiency of the combustion process [10,11]. However, biomass ash (except rice husk ash and sugarcane bagasse ash, which are rich in SiO_2_) has a low inorganic concentration, so utilizing such residues is difficult. Rice husk ash contains a large amount of SiO_2_ [12,13,14,15,16,17,18,19,20,21] and is an effective precursor for binder systems (cement and alkali activator-based binders) [22,23,24,25,26].

Aqueous alkaline solutions promote the dissolution of alumina and silica from biomass ash. Residual unburned carbon in biomass ash complicates its usage in cementitious materials, and higher carbon content is associated with increased adsorption of air-entraining admixtures and higher water consumption [27,28]. In many developing countries in Asia and Africa, biomass incineration for energy generation has increased significantly in recent years, resulting in large quantities of biomass ashes [12]. Biomass fly ash (BFA) has traditionally been utilized as a mineral fertilizer in agriculture [29], but commercial utilization of biomass ash is not so widely reported. Due to the expected growth in this waste, it is vital to investigate its potential uses. Most biomass ash produced in thermal power plants is now disposed of in landfills or recycled in agricultural fields or forests with little or no oversight [30]. However, given that the cost of disposing of biomass ashes is increasing and that biomass ash quantities are expanding globally, it is critical to developing a solution for the sustainable management of such waste. In this regard, some researchers have considered using biomass ashes from wood burning as a concrete substitute [28,31].

Research [32,33,34] proves that biomass ashes can be used in road construction. On the other hand, biomass ashes worsen the rheological behaviour and compressive strength of concrete [30,31,35,36], depending on the pozzolanic nature of biomass ashes and the higher organic content of 20–25% [37]. It has also been pointed out that biomass ashes affect the apparent density, water absorption of concrete [30], and the continuous degradation of compressive strength of specimens when the amount of biomass ash is increased.

One of the more environmentally friendly solutions for recovering biomass ashes is to use them as binders in alkali-activated substances. Due to almost similar binding characteristics, alkali-activated materials (AAM) are a viable alternative to Portland cement [38,39,40]. AAM has attracted much interest in recent years because of its satisfying early compressive strength, low permeability, and chemical resistance. The use of Na_2_CO_3_ solutions to activate slags, which have high calcium amounts, has been investigated extensively, while its use in the activation of fly ash is significantly less [39]. Using mixed sodium hydroxide-carbonate activating solutions produces a porous, poorly reacted product, although it is uncertain what role the carbonate component of the activating solution plays in activated fly ash [41]. According to researchers, fly ash (Class F) is less reactive than slags and therefore requires stronger alkalinity than Na_2_CO_3_ solution.

However, the practical utilization of traditionally used NaOH and Na_2_SiO_3_ as activators has been hindered by sustainability concerns and handling issues due to the high alkalinity [42,43]. In addition, hydroxides are made from the electrolysis of chloride salts, which consume a lot of energy and emit CO_2_. Alkali silicates are made by melting sand with carbonates at 1100–1200 °C and then dissolving them under high pressure at 140–160 °C [44,45]. In addition to its high prices, using NaOH as an activator has the problems of quick setting and drying shrinkage [46,47,48,49]. Although much attention has been paid to fly ash/slag mix activated with alkali hydroxides/silicates, alternate activation systems still need to be found to sort out the efflorescence problem associated with NaOH and its handling issue due to the high alkalinity [42,43]. The use of other types of activators, such as Ca(OH)_2_, Na_2_SO_4_, Na_2_CO_3_, CaO, and MgO, offers a promising option for reducing the environmental impact of AAM [50,51,52,53,54,55,56].

As an alternative, Na_2_CO_3_ can be used as an activator to create long-lasting AAM systems in less alkaline environments and with lower drying shrinkage [57,58,59]. Na_2_CO_3_ possesses low cost and commercial availability and helps reduce the pH of alkaline activator solution (AAS). Despite these benefits, the use of Na_2_CO_3_ as an activator in AAS systems has been infrequently reported, owing to low pH limitations resulting in long setting times and limited strength development [60,61]. The reaction kinetics of AAM can be enhanced to some extent by introducing higher concentrations of hydroxides [62,63,64,65], sodium silicate [66], or blending with reactive admixtures such as CaO [67]. Nonetheless, all these approaches are ineffective, expensive, or have negative effects. For example, by adding NaOH to the activator, samples can achieve a 1-day compressive strength of about 5–7 MPa, with almost no additional strength development after 7 days of curing [64,68]. A prolonged reaction process was reported in [52,69], whereby demolding took approximately 3–5 days, depending on the composition. The subsequent reaction is cyclic with a buffered alkaline environment regulated by CO_3_^2−^ anions and maintained by continuous CaCO_3_ dissolution [70]. According to the study [52], the precipitation of CaCO_3_ and consumption of CO_3_^2−^ anions produced by the activator are mostly responsible for the delayed reaction process of Na_2_CO_3_-activated slag. However, on the contrary, another study reported that compositions activated by Na_2_CO_3_ show satisfactory setting time and compressive strength during 1–3 days of curing, reaching 5–25 MPa [71,72]. The activation potential of activators might be arranged in the following order [73]: Na_2_SiO_3_ > NaOH > NaOH + Na_2_CO_3_ > KOH.

It is pointed out that hydroxides accelerate the reaction of Na_2_CO_3_-activated slag by removing the CO_3_^2−^ anions, resulting in a significant rise in pH, and the samples can be hardened within 24 h [74]. The 7-day compressive strength of ground granulated blast-furnace slag (GGBS) pastes activated by NaOH/Na_2_SiO_3_ is three times that of Na_2_CO_3_-activated GGBS pastes [69,75]. The Ca^2+^ ions released from the dissolved GGBS interact with CO_3_^2−^ anions and create carbonates (e.g., calcite and gaylussite). If this reaction is prolonged because of the solution’s low pH, the dissolution time of the silicate species is prolonged, as is the setting time [69,76]. The consumption of CO_3_^2−^ anions releases hydroxide ions, which raise the pH of the liquid phase, forcing the silicate species to dissolve and C-(A)-S-H to form [77].

In the early stage, the calcium carbonate and sodium-calcium carbonate phases are formed as the Ca^2+^ ions released from slag react with the CO_3_^2−^ anions of the dissolved Na_2_CO_3_ in the pore solution. Meanwhile, the dissolution of the slag releases silicate and aluminate ions, which react with the sodium of the activator to form zeolite NaA [78]. Additionally, synthetic C-S-H seeds [79] or MgO [80,81,82,83,84,85] are suggested for activation reaction improvement. Such additives provide extra nucleation sites, higher pH, additional formation of hydration products, and early strength gain. Compared with Portland cement-based materials, the application of using biomass ashes to make AAM [30,31,35,36,37] has excellent potential, and the use of low-cost unprocessed BFA for producing AAM has not been described before.

This research aims to study the characteristics of the used BFA and the development of BFA-based AAM. Therefore, experimental research was carried out to investigate the effects of the Na_2_CO_3_/Na_2_SiO_3_ (C/S) ratio and curing temperature (40 °C and ambient temperature) on the fresh-state properties (e.g., setting time), structure formation, product synthesis, and physical-mechanical properties of BFA-based AAM pastes. The use of unprocessed BFA to make AAM can reduce production costs. Moreover, using less alkaline Na_2_CO_3_ as an activator benefits the development of environmentally friendly products. The findings of this study contribute to the applicability of difficult-to-recycle waste materials such as BFA and the development of sustainable BFA-based AAM. The study results could also encourage the construction industry to use such materials to reduce the negative environmental impact of their storage.

## 2. Materials and Methods

### 2.1. Raw Materials

The main precursor in this research is BFA from the biomass power plant. The wooden chips of pine trees were used as biomass. On average, the coniferous wood contains (48–56%) cellulose, (26–30%) lignin, and (23–26%) hemicelluloses containing (10–12%) pentosans and about 13% hexosans. When wood is heated at an increasing temperature, the processes of its drying, pyrolysis and gasification, and accompanying combustion proceed sequentially. At a temperature of 250–350 °C, the wood begins to decompose into components under the effect of high heat. As a result of pyrolysis (thermal destruction) of wood, volatile substances are released. The products of wood pyrolysis are mainly tar, coal, and low molecular weight gases, and large amounts of carbon monoxide and dioxide (CO and CO_2_) are also released. Pine burning is characterized by a low combustion temperature of about 610–630 °C and the formation of smoke and soot.

The chemical composition of the BFA is presented in Table 1. As can be observed, the main oxides presented in the BFA are CaO (31.50%) and SiO_2_ (22.91%), and carbon occupied 20.40% of the composition. This carbon content is apparently due to the incomplete combustion process of the wood [86]. It is common for BFA to contain such a large amount of carbon because it is difficult to maintain a smooth combustion process in bio-boilers. In the BFA, the alkalis are presented as K_2_O (4.003%) and Na_2_O (0.259%), and the BFA contains a significant amount of MgO (3.574%). The contents of chlorides and sulfates are not high, while P_2_O_5_ content is noticeable higher. The presence of heavy metals such as Cr, Cu, Ni, and Zn in the BFA is not high. The most critical components in wood ash are CaO, SiO_2_, Al_2_O_3_, Fe_2_O_3_, and MgO, as they react in the existence of moistness to create bonding agents.

**Figure 1:**
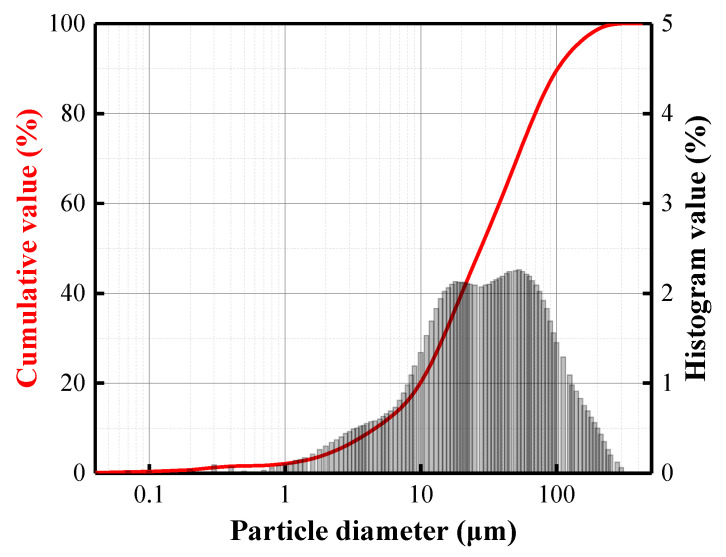
Particle size distribution of the BFA.

It is known that fly ash must meet the standard requirements (BS EN 450-1:2012) to be used as a substitute for cement. The chemical composition of the BFA was compared with the requirements for fly ash. The total content of (SiO_2_ + Al_2_O_3_ + Fe_2_O_3_) in the BFA is almost three times lower, and the LOI is 2.5 times higher than specified for fly ash in the standard. The BFA has a bulk density of 683 kg/m^3^, a specific surface area of 493 m^2^/kg, and an average particle size of 43.26 μm (Figure 1).

**Figure 2:**
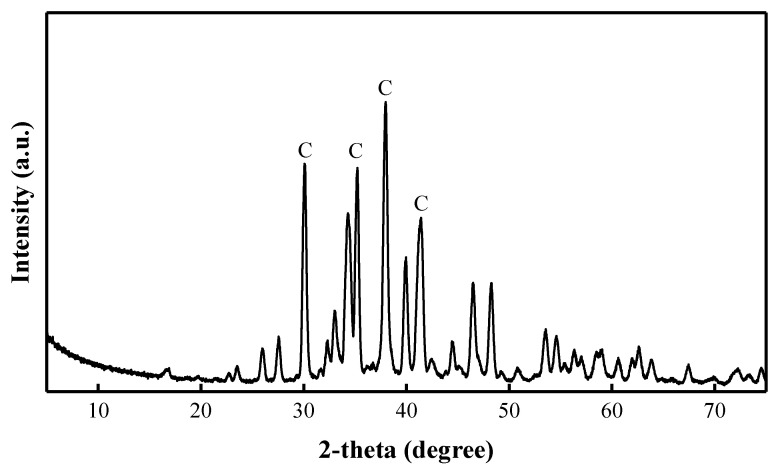
XRD pattern of Na_2_CO_3_ (C: Na_2_CO_3_).

**Figure 3:**
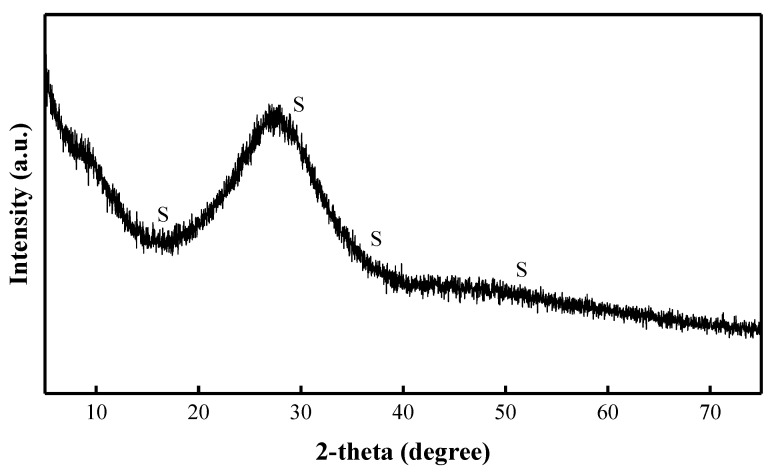
XRD pattern of Na_2_SiO_3_ (S: Na_2_SiO_3_).

Two components were used as an alkaline activator solution: Na_2_CO_3_ solution and sodium silicate (Na_2_SiO_3_·nH_2_O, H_2_Na_2_O_4_Si) solution. Na_2_CO_3_ solution (30 wt%) was prepared by dissolving Na_2_CO_3_ crystalline powder with water. Figure 2 shows the XRD pattern of Na_2_CO_3_. Na_2_SiO_3_ solution concentration was 50 wt% with a density of 1.37 kg/m^3^, and the molar ratio of SiO_2_ to Na_2_O ratio was 3.22, and the boiling point of Na_2_SiO_3_ is 100 °C. Figure 3 shows the XRD pattern of Na_2_SiO_3_.

Polycarboxylate-based superplasticizer (SP) with 27% dry matter content of the solution, with a molecular weight of 51 g/mol, was used to maintain the same consistency in these samples, and the SP was used in a liquid state.

### 2.2. Paste Design and Sample Preparation

The BFA-based AAM pastes were prepared according to the mix design in Table 2. To study the effect of the C/S ratio on the structure formation of BFA-based AAM pastes, the AAS was designed with different C/S ratios. The Na_2_CO_3_/Na_2_SiO_3_ solute ratio varied from 0.40 to 1.20, while the solution ratio varied from 0.67 to 2.00. The AAS and the SP solution were constant at 25 wt% and 5.71 wt% of the BFA, respectively. The water/BFA ratio of these samples was constant at 0.35. The water in the system includes water in the Na_2_CO_3_-Na_2_SiO_3_ solution, SP solution, and additional water.

**Figure 4:**
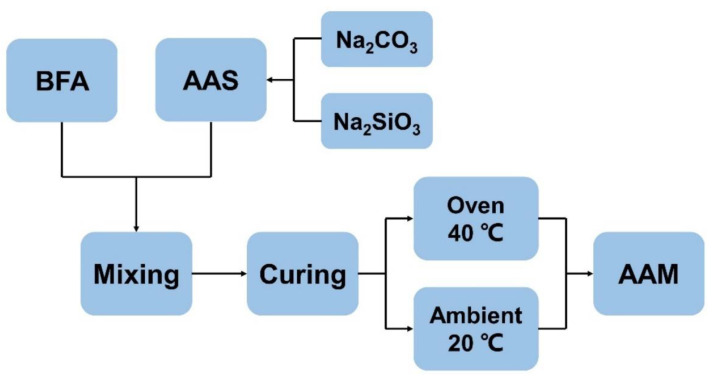
Schematic of BFA-based AAM paste preparation.

After mixing, the fresh AAM pastes were cast in 160 × 40 × 40 mm steel molds and compacted on the vibrating table for 20 s. The BFA-based AAM paste molds were divided into two groups: one group was kept at an ambient temperature of 20 ± 2 °C for 24 h and then demoulded and kept at the same temperature; another group of samples was kept at 40 °C in the oven (Figure 4). After 24 h, samples were demolded and further kept in the oven at 40 °C for all testing times.

### 2.3. Test Methods

BFA powder analysis was performed using a high multichannel performance sequential Wavelength Dispersive X-ray Fluorescence (WD-XRF) spectrometer (AXIOS-MAX, Panalytical, Eindhoven, Netherlands). The WD-XRF system operated for 1440 s. The quantitative analysis was performed using “Omnian” software, and corresponding standards were used for a standard-less analysis. X-ray diffraction (XRD) patterns of the studied BFA and composites (CS-0.40-40, CS-0.75-40 and CS-1.20-40) were measured using an X-ray diffractometer, SmartLab (Rigaku, Tokyo, Japan), equipped with an X-ray tube with a 9 kW rotating Cu anode. The measurements were performed using Bragg–Brentano geometry with a graphite monochromator on the diffracted beam and a step scan mode with a step size of 0.02° (in 2θ scale) and a counting time of 1 s per step. The measurements were conducted in the 2θ range of 5–75°. Phase identification was performed using the software package PDXL (Ver. 2.8, Rigaku) and the ICDD powder diffraction database PDF4+ (2021 release). Mineral content was estimated using Reference Intensity Ratio (RIR) method.

Thermogravimetry/differential thermal analysis (TG/DTA) curves of the BFA were registered with a Linseis STA PT-1600 thermal analytical instrument up to 1000 °C (with a temperature rise rate of 10 °C/min; the air was used as the heating environment; the weight of the specimens was (50 ± 5) mg). According to standard NF P18-513 and [87], Chapelle’s method was used to determine the pozzolanic activity of the BFA. The hydraulic activity of the BFA was calculated based on the standard requirements (BS EN 197-1:2011). The hydraulic index K_3_ was calculated based on the chemical composition of the studied BFA and using calculation methods presented in [88] and [89]. The hydraulic index K_3_ is defined as the ratio of basic oxides to acidic oxides: (%CaO + %MgO + %Al_2_O_3_)/(%SiO_2_). Values of K_3_ above 1.0 indicate good hydraulic properties.

The effect of different concentrations of the prepared Na_2_CO_3_ solutions and Na_2_SiO_3_ solution on the electrical conductivity (EC) and pH values were performed with the MPC 227 instrument manufactured by “Mettler-Toledo” (Columbus, OH, USA, pH electrode InLab 410, measuring accuracy 0.01; EC electrode InLab 730, measuring range 0–1000 mS/cm). The EC and pH values of the prepared activator solutions and alkali-activated BFA pastes with different C/S ratios (Table 2) were measured at 20 °C.

The structure formation of the samples cured at 40 °C and 20 °C was evaluated using the ultrasonic pulse velocity (UPV) method using the tester Pundit 7. The sample was placed between two ultrasonic transducers (transmitter and receiver) operating at 54 kHz. The transducers were pressed against the samples at two strictly opposite points, and Vaseline was used to ensure good contact. The ultrasonic pulse velocity (*V*) was calculated using Equation (1):

(1) $$V=\frac{l}{\tau }\cdot 10^{6}$$

where *l* is the distance between the cylindrical heads and *τ* is the time of pulse spread.

The compressive strength, bulk density, water absorption, and porosity of the samples cured at 40 °C and 20 °C during 28 days were determined according to standard LST EN ISO 1927-6:2013.

## 3. Results and Discussion

### 3.1. Parameters of BFA

**Figure 5:**
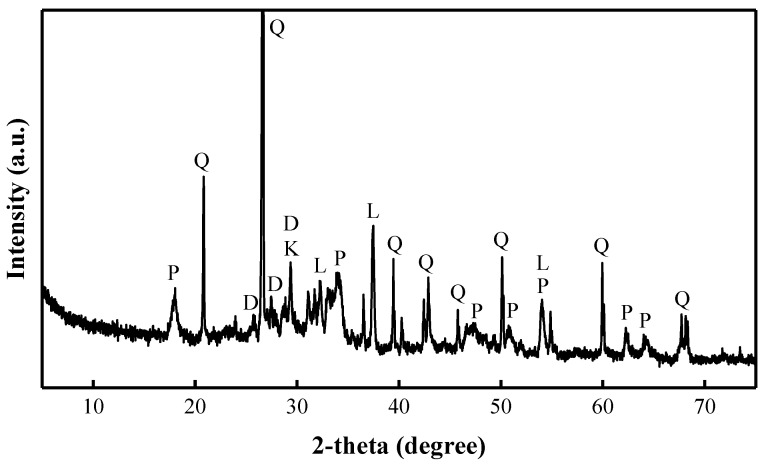
XRD pattern of the BFA (Q: quartz; D: dicalcium silicate; P: portlandite; L: lime; K: calcite).

According to the X-ray phase analysis (Figure 5), the BFA consists of quartz—52.0%, dicalcium silicate—19.7%, portlandite—15.0%, lime—9.20%, and calcite—3.80%.

**Figure 6:**
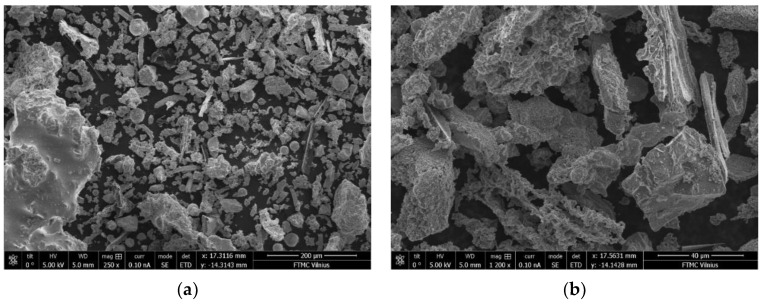
SEM images of the BFA. Magnification: (**a**) 250 and (**b**) 1200.

The SEM images of the BFA are presented in Figure 6. Most BFA particles have irregular shapes, while other ash particles show approximately spherical shapes. The parts of unburned wood or soot particles are sufficiently noticeable.

**Figure 7:**
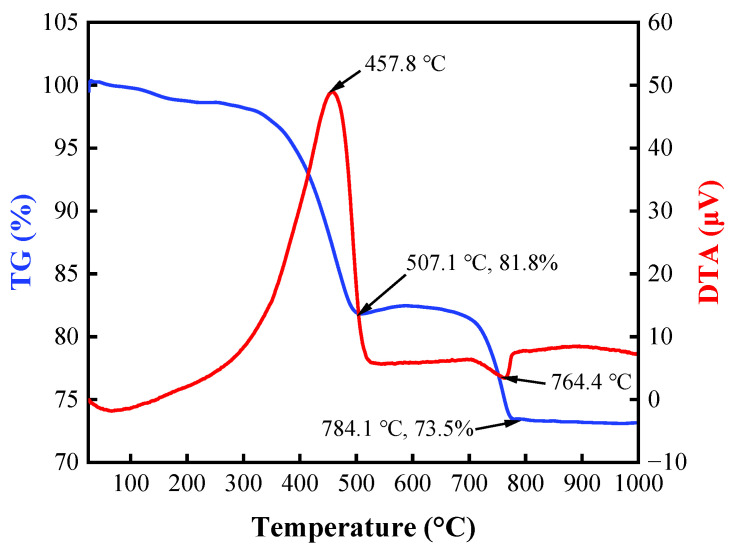
TG/DTA curves of the BFA.

It can be observed that the TG/DTA curves of the BFA have one exothermic effect in the temperature interval of 300–520 °C and two endothermic effects in the temperature intervals of 20–200 °C and 680–780 °C (Figure 7). In the temperature interval of 20–200 °C, the unbound water and a part of bound water are removed with a mass loss of approximately 1.8%. There is a clearly visible exothermic peak in the temperature range of 300–520 °C, and the exothermic process can be attributed to the combustion of soot and organic matter (unburned wood aggregate) [90]. During the combustion of wood, some decomposition takes place at temperatures as follows: hemicellulose (200–260 °C); cellulose (240–350 °C); and lignin (280–500 °C) [91,92]. Hemicellulose and cellulose are characterized by fast thermal decomposition, while lignin is more resistant, and during its pyrolysis, carbon layers on the lignin surface are formed. During this burning process, mass loss is the highest. Although portlandite appeared in the XRD test, the decomposition of this mineral is hindered by the exothermal effect of unburned wood aggregate. The endothermal effect in the temperature range of 680–780 °C is attributed to the decomposition of calcite.

The pozzolanic activity of the BFA is 45.7 mg CaO/g. It indicates that the BFA is not rich in amorphous SiO_2_, which can react with the Ca(OH)_2_ to create additional hydration products, such as C-S-H, which improves the physical and mechanical properties of the cementitious system. SiO_2_ is usually formed at high temperatures during the incineration of biomass. However, in our research, the BFA was collected from a low-temperature combustion process and is characterized by properties the same as referred to in research [93,94]. In the study [87], after a series of experiments, the following pozzolanic activities of biomass fly ash were determined: sugar cane biomass fly ash—279 mg CaO/g, rice husk biomass fly ash—622 mg CaO/g, wood biomass fly ash—269 mg CaO/g.

The ability of a material to set and harden when mixed with water and produce hydration products during the hydration process is referred to as hydraulic activity. The contents of SiO_2_ and CaO determine the hydraulic activity, and the standard requirement (BS EN 197-1:2011) for hydraulic material is that the mass ratio of CaO/SiO_2_ is not less than 2. Based on the data in Table 1, the hydraulic activity of the BFA is 1.375. Hydraulic index K_3_ is calculated based on the chemical composition of the studied BFA, defined as the ratio of basic oxides to acidic oxides: (%CaO + %MgO + %Al_2_O_3_)/(%SiO_2_) [88]. Based on the data in Table 1, the hydraulic index K_3_ of the studied BFA is 1.645.

Overall, it can be concluded that the BFA has low hydraulic properties and is reasonable for use as a precursor in alkali-activated materials.

### 3.2. Activation Solution Characterization

The tests were carried out to describe how Na_2_SiO_3_ solutions and different concentrations of Na_2_CO_3_ in solutions affect the values of EC and pH (Table 3). According to the results, the highest pH is Na_2_SiO_3_ solution; for Na_2_CO_3_ solutions, the pH varies a little. The concentration of Na_2_CO_3_ affects the EC value of the solution, as the EC value depends on the number of ions in the solution. The EC value in the 10% Na_2_CO_3_ solution is 18% lower than in the 20% Na_2_CO_3_ solution. However, when the concentration of Na_2_CO_3_ in the solution was increased to 30%, the EC value decreased to 79.2 S/m. This reduction in EC value is assumed to occur due to supersaturation of the solution, as also noted in studies [87,93,94].

**Figure 8:**
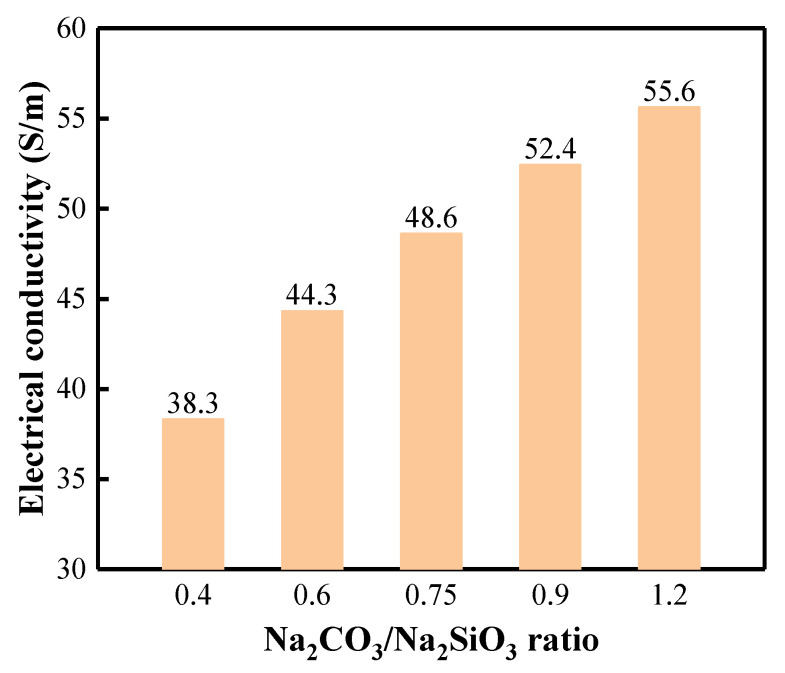
EC of BFA-based AAM pastes with different C/S ratios.

The EC values of BFA-based AAM pastes with different C/S ratios are presented in Figure 8. The research results show that the number of ions in the solution increases with the increased amount of Na_2_CO_3_. The EC value gradually increases by increasing the C/S ratio in the paste. When the C/S ratio triples, the EC value increases by 45.2%. However, the dissolution of BFA minerals and the penetration of ions into the solution were delayed, and the EC values increased slowly in pastes with larger C/S ratios.

### 3.3. Initial and Final Setting Times of BFA-Based AAM Pastes

**Figure 9:**
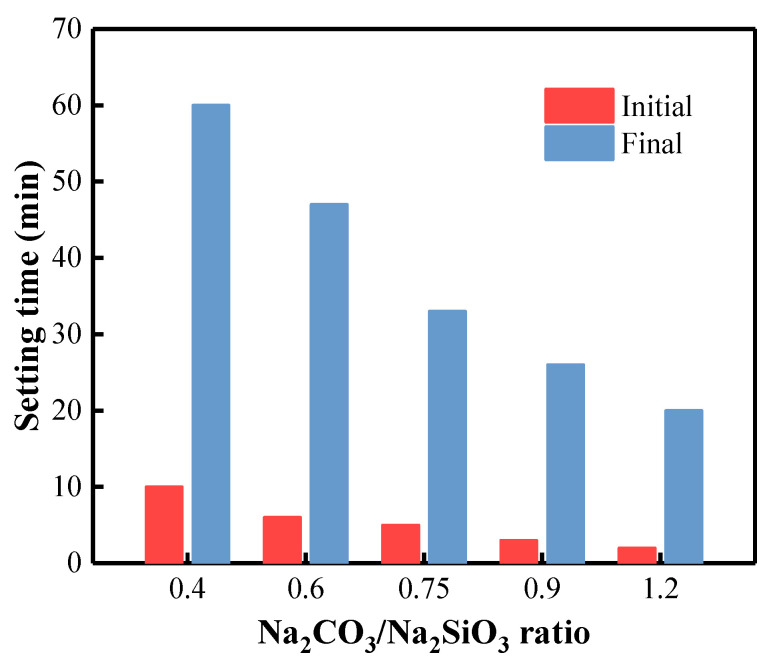
Initial and final setting times of BFA-based AAM pastes with different C/S ratios.

To choose the most appropriate C/S ratio, setting time tests were performed, including the initial and final setting times of prepared BFA-based AAM pastes (Figure 9). Initial setting time results show that CS-0.40 paste, which possesses the lowest EC, is characterized by the longest initial setting time. As the C/S ratio increases, the initial setting time decreases, and the interaction between the AAS with higher Na_2_CO_3_ content and the BFA with high lime content starts faster. When the Na_2_CO_3_ content in the BFA-based AAM paste is the highest, the final setting time is one-third of that with the lowest Na_2_CO_3_ content.

Researchers have studied the workability of mixtures containing different amounts of alkali [62] and found that the initial flow of the manufactured mortar decreased slightly with increasing alkali content. However, it was pointed out that higher doses of Na_2_CO_3_ lead to faster sintering reaction rates [52,95]. The availability of CO_3_^2−^ anions in the pore solution also substantially affects the reaction kinetics [52]. As Na_2_CO_3_ increases, more Ca^2+^ ions can be dissolved at the early stage and precipitated with CO_3_^2−^ anions, thus speeding the reaction and the setting of AAM. Similar results have emerged in the studies [52,69]. It was pointed out that the duration of the induction period of Na_2_CO_3_ activated slag cement with an alkali concentration of 3 Na_2_O% is 1.6 times that with an alkali concentration of 8 Na_2_O%. The delayed reaction of slag cement activated by lower concentrations of Na_2_CO_3_ can be explained by the initial chemical precipitation of CaCO_3_.

### 3.4. Density of BFA-Based AAM Pastes

**Figure 10:**
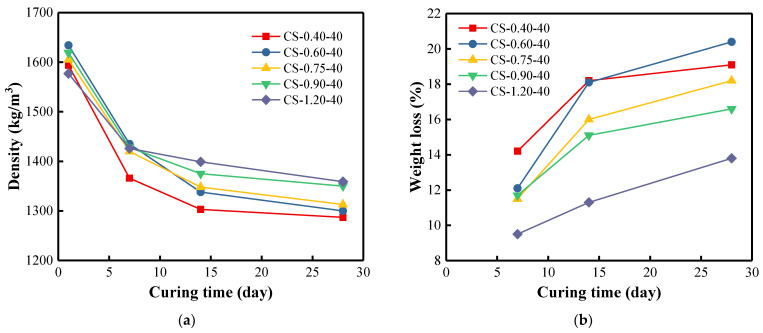
(**a**) Density of BFA-based AAM pastes cured at 40 °C for 28 days; (**b**) weight loss of samples in % during 28 days.

Densities of the samples cured at 40 °C (Figure 10a) decreased rapidly for the first 7 days and then leveled off gradually. A reduction in weight loss of 14.2% to 9.5% after 7 days of curing can be observed as the C/S ratio increases from 0.40 to 1.20 (Figure 10b). After 28 days of curing, the weight loss is in the range of 20.4–13.8%. It can be clearly observed that by increasing the C/S ratio, the weight of samples is reduced less. As the C/S ratio increases visibly, the amount of free water that can be evaporated decreases with its participation in the synthesis reactions. EC studies of the pastes confirm this reasoning (Figure 8), showing that the EC value increases with increasing the C/S ratio. As was pointed out earlier, an increase in CO_3_^2−^ anions promote Ca^2+^ ions dissolution and new phase formation, which involve water and prevent evaporation.

**Figure 11:**
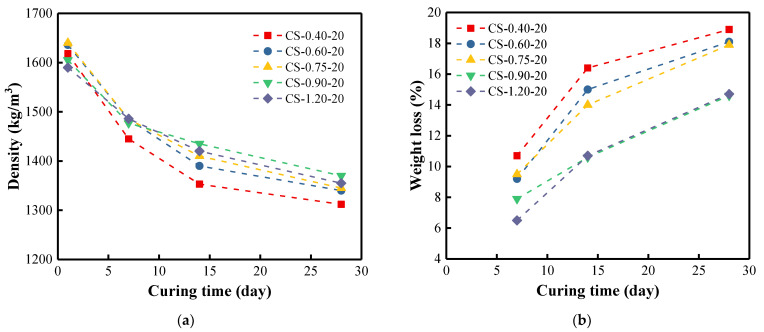
(**a**) Density of BFA-based AAM pastes cured at 20 °C for 28 days; (**b**) weight loss of samples in % during 28 days.

The same trend was observed for the samples cured at 20 °C (Figure 11). However, the densities of the samples cured at 20 °C decreased significantly during 14 days, and the weight losses were less than that of the samples cured at 40 °C. The sample with the smallest C/S ratio always showed the smallest density whenever it was at 40 °C or 20 °C. The higher the curing temperature, the faster the structure evolution during the BFA-based alkali activation-synthesis process. The same trend was observed in research for samples cured at ambient temperature [96,97]. The study concluded that the density of the cured samples decreased slightly by about 2% in the first few weeks, but remained almost constant thereafter. In our case, the weight losses were more pronounced because of using the different activators, lower curing temperature, and high carbon content of BFA. The density tests were supplemented by UPV testing of the samples.

### 3.5. UPV of BFA-Based AAM Pastes

**Figure 12:**
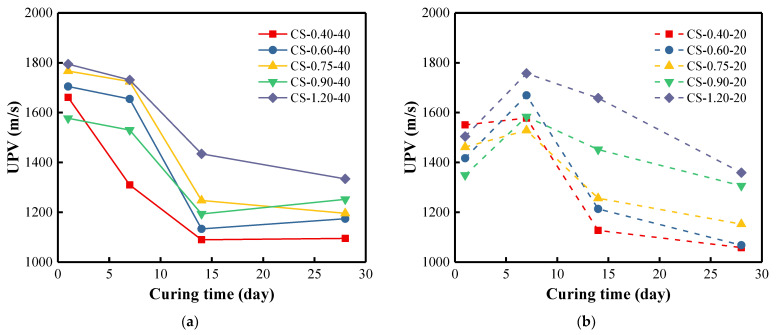
UPV of BFA-based AAM pastes cured at (**a**) 40 °C and (**b**) 20 °C for 28 days.

Density trends are reflected in the structure formation of BFA-based AAM pastes (Figure 12). At the curing temperature of 40 °C, the UPV values of the samples generally showed a downward trend within 14 days, and then the UPV values changed slightly until 28 days. It seems that the structure formation was generally completed in this period. UPV values were consistently lower in the samples with lower C/S ratios. The UPV values of the sample with the smallest C/S ratio decreased the fastest and then leveled off. It is shown that the more Na_2_CO_3_ in the AAS, the faster the solidification of the AAM paste. At the curing temperature of 20 °C, various increases in UPV values were observed for the structure formation of the samples after 7 days. Further structure formation was slow, and the UPV values decreased slightly. It can be concluded that higher curing temperatures accelerate the AAM synthesis.

### 3.6. Compressive Strength of BFA-Based AAM Pastes

**Figure 13:**
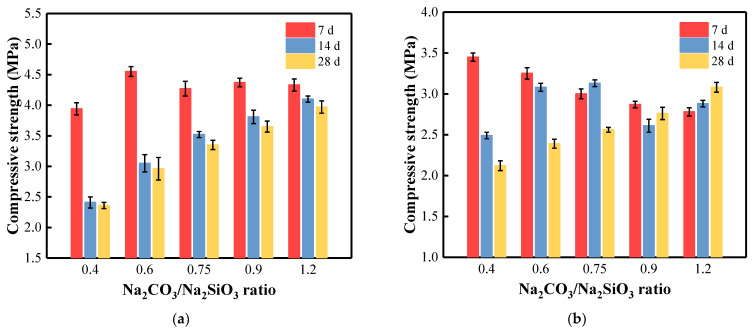
Compressive strength of AAM pastes with different C/S ratios cured at (**a**) 40 °C and (**b**) 20 °C.

Figure 13a presents the compressive strength of samples with different C/S ratios cured at 40 °C. After curing for 7 days, the compressive strength increased with the increase in the C/S ratio, indicating that the higher the alkali content, the better the mechanical properties [62,98]. Depending on the amount of Na_2_CO_3_, the compressive strength of the samples at 7-day curing ranged from 3.94 to 4.55 MPa (Figure 13a). However, when the curing time reached 28 days, the strength of the samples showed a decreasing trend, from 2.36 MPa (the lowest C/S ratio) to 3.97 MPa (the highest C/S ratio). This trend is mainly observed when the C/S ratio is lower. From 7 to 28 days, the sample CS-0.40-40 showed the largest strength loss with a 40% decrease. Moreover, the sample with the smallest C/S ratio always showed the smallest density at 40 °C. For the sample CS-1.20-40 with the highest C/S ratio, the compressive strength decreased the least, by only 8.3%.

Overall, the compressive strength of the samples cured at 40 °C was higher than that of the same composition cured at 20 °C. For samples cured at 20 °C, the sample (CS-0.40-20) with the smallest C/S ratio showed a sharp decrease in compressive strength over the curing time (from 3.45 to 2.12 MPa, decreased by 38.6%). However, with increasing the C/S ratio, the compressive strength of CS-1.20-20 increased slightly (from 2.78 to 3.08 MPa, increased by 10.8%) during the curing time (Figure 13b). It can be explained by the fact that dicalcium silicate, one of the key minerals of the BFA, dissolved better in AAS at a higher temperature than in the pastes activated at ambient temperature [99]. Researchers pointed out that amorphous hydrated C-(A)-S-H-like gels with aluminum and sodium ions were generated during structure formation. As a result, these reaction products occupied the cavities and pores, increasing the samples’ mechanical strength.

Several research results [100,101] can be presented about using wastes from various organic sources in geopolymers. As pointed out in [100], greener geopolymers have been developed using agricultural and industrial wastes such as rice husk, rice husk ash, metakaolin, ground granulated blast furnace slag, and palm oil fuel ash, activated by different ratios of activators (Na_2_SiO_3_/NaOH). The results showed that the compressive strength of geopolymers was directly proportional to the ratio of the alkaline solution, while the compressive strength of the samples ranged from 0.8 to 2.8 MPa and up to 4.9 MPa. The same low strength of 4–6 MPa was reported in [96], when only biomass fly ash or fly ash was used in the alkali-activated materials composition. In the synthesized one-part geopolymers, consisting of fly ash, slag, and hydrated lime, the 28-day compressive strength of the fly-ash-based samples cured at ambient temperature reached 5.2 MPa when activated with Na_2_SiO_3_ [101]. Overall, a higher curing temperature and a higher C/S ratio can improve the strength under certain conditions.

### 3.7. Water Absorption of BFA-Based AAM Pastes

**Figure 14:**
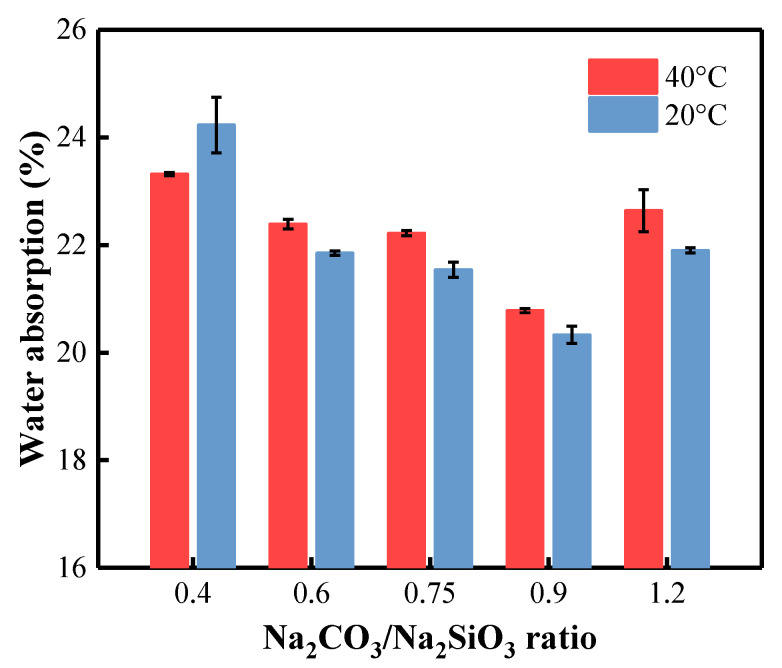
Water absorption of AAM pastes with different C/S ratios cured at 40 °C and 20 °C.

By comparing the density and water absorption of AAM samples, they both show some significant correlations. Overall, the water absorption of the samples cured at 40 °C is slightly higher than that of the same composition cured at 20 °C (Figure 14). After curing at 40 °C and 20 °C for 28 days, the sample CS-0.40 with the smallest density displayed higher water absorption. When the C/S ratio in the AAM increases from 0.40 to 0.90, the water absorption decreases from 23.32% to 20.78% for samples cured at 40 °C and from 24.23% to 20.33% for samples cured at 20 °C. The presence of BFA in AAM increases the water absorption of the samples [86]. The apparent density of BFA-based composite activated by Na_2_CO_3_ varies between 1.58–1.6 kg/m^3^, and water absorption varies between 20–22%. In our case, this could be due to the high content of unburned carbonized organic matter, which actively adsorbs water due to its highly porous structure [102,103]. The densities of our samples are significantly lower (1.27–1.37 kg/m^3^), which results in higher water absorption values.

### 3.8. XRD Patterns of BFA-Based AAM Pastes

**Figure 15:**
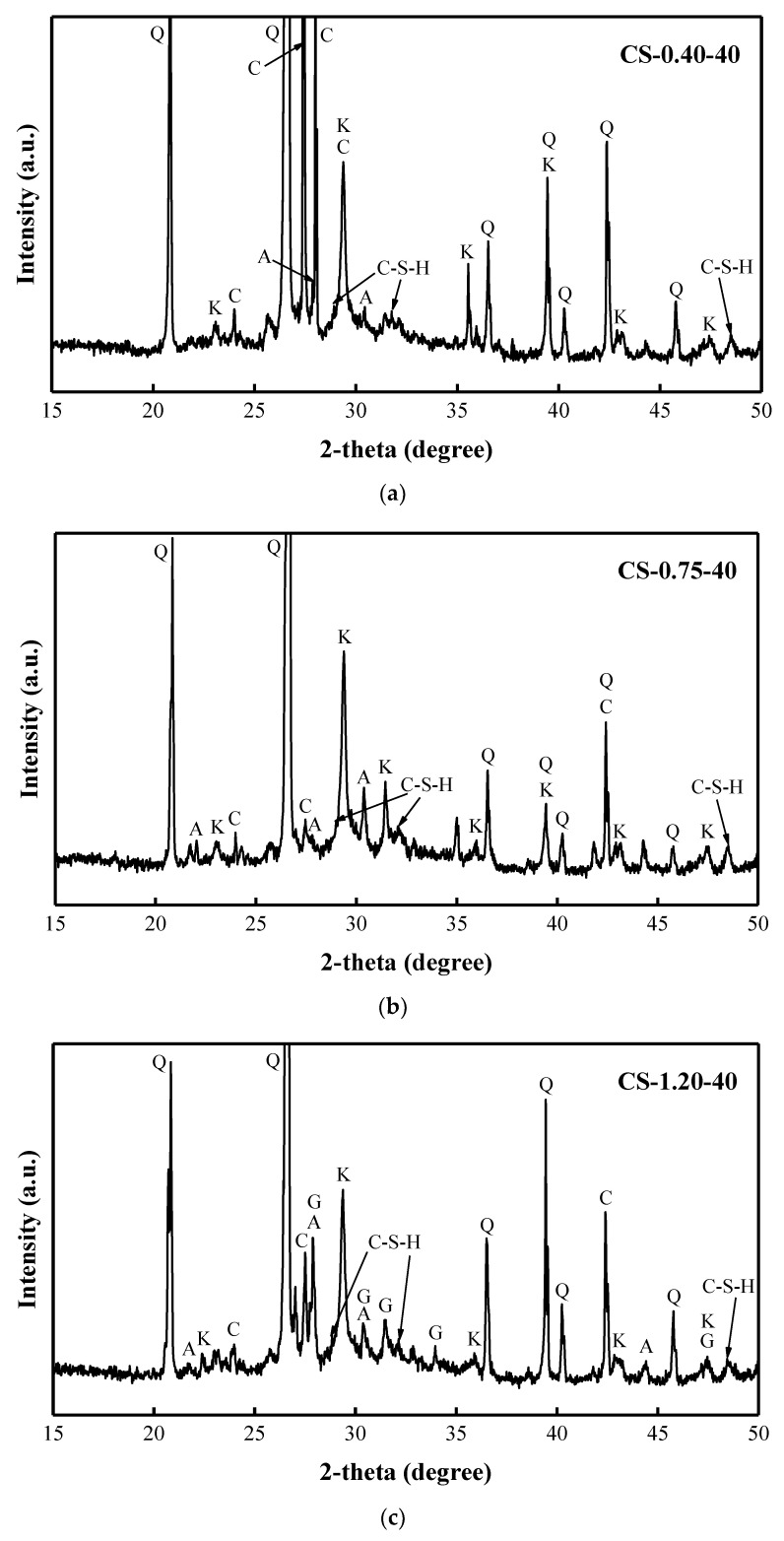
XRD patterns of AAM pastes cured at 40 °C for 28 days: (**a**) CS-0.40-40; (**b**) CS-0.75-40; (**c**) CS-1.20-40 (Q: quartz; K: calcite; G: gaylussite; A: anorthoclase; C: Na_2_CO_3_; C-S-H: C-S-H gel).

Samples cured at the higher temperature of 40 °C show higher compressive strength results than samples cured at 20 °C. Therefore, the reaction products of the samples cured at 40 °C with the C/S ratio of 0.40, 0.75, and 1.20 were characterized by XRD tests (Figure 15). After curing for 28 days, the main mineral in the AAM pastes of all compositions is quartz, which is also the main mineral of the BFA. The mineralogical composition of Na_2_SiO_3_ showed a diffuse broad hump in the range of 23–35° 2θ (Figure 3) [62]. The most intensive peaks of Na_2_CO_3_ were observed at 28–46° 2θ (Figure 2). A diffuse broad hump attributed to the amorphous phase has been identified in the diffractograms of all samples. It was assumed that due to the high pH in the AAS, the formation of the crystalline phase from the zeolitic germs formed in the samples was slow, and thus an amorphous (C-S-H/C-A-S-H) phase was produced [41].

The reaction products of the sample with a C/S ratio of 0.40 are calcite and C-S-H. Additionally, some Na_2_CO_3_ and anorthoclase have been identified. It should be noted that the most intense peaks of Na_2_CO_3_ were observed in this composition. As the C/S ratio increases, the intensity of the Na_2_CO_3_ peak decreases. The reaction products in the sample with a C/S ratio of 0.75 are the same. The growth of gaylussite was observed in the sample with a C/S ratio of 1.20. In addition, the above minerals Na_2_CO_3_, C-S-H, and anorthoclase have all been identified. These reaction products (calcite, C-S-H, and gaylussite) control the compressive strength growth of the AAM pastes.

Along with the identified reaction products (calcite and gaylussite) in the composition with the same activator, the researchers also identified poorly crystalized C-(A)-S-H gels [62]. According to the researchers, Ca^2+^ ions are available and quickly precipitated with CO_3_^2−^ anions generating calcite and gaylussite. The transformation of initially precipitated CaCO_3_ to other phases is the key to the reaction development [62]. The crystalline phases, such as quartz, in the raw BFA remained unaltered after the activation of BFA pastes. The same observations were reported in the research [104].

### 3.9. TG/DTA Test of BFA-Based AAM Pastes

**Figure 16:**
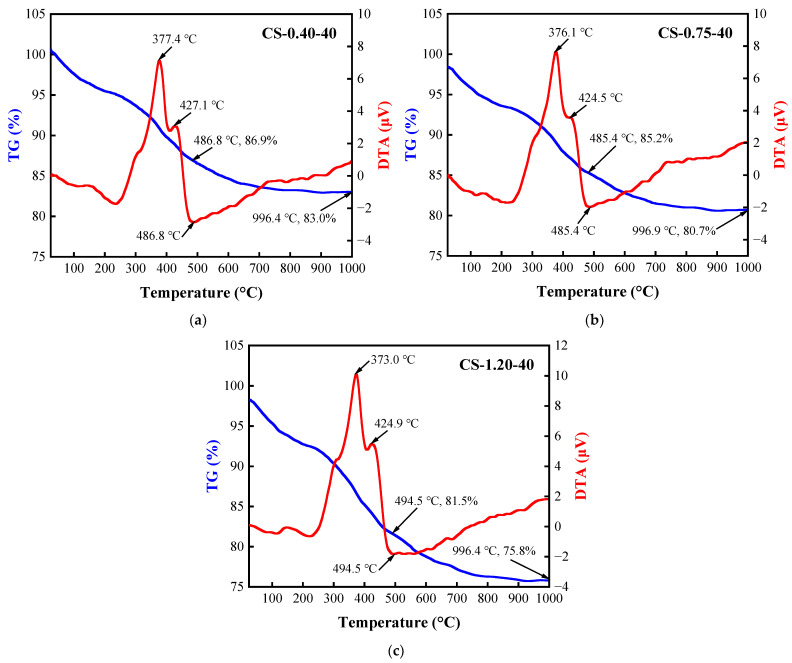
TG/DTA curves of AAM pastes cured at 40 °C for 28 days: (**a**) CS-0.40-40; (**b**) CS-0.75-40; (**c**) CS-1.20-40.

The TG/DTA curves of BFA-based AAM pastes (C/S ratios of 0.40, 0.75 and 1.2) cured at 40 °C for 28 days show two endothermic effects in the temperature intervals of 20–250 °C and 680–760 °C, and three exothermic effects in the temperature intervals of 280–320 °C, 330–380 °C, and 400–500 °C (Figure 16). In the temperature interval of 20–200 °C, the unbound and partially bound water was removed from the samples. At the same temperature interval (160–200 °C) from which the decomposition of the C-S-H undergoes [105], dehydration of gaylussite in the temperature interval of 200–250 °C was observed [106,107,108]. These findings are supported by the XRD results (Figure 15).

The mass loss of the samples in the temperature range of 20–250 °C increases from 5.8% to 7.2% and 8.5% when changing the C/S ratio from 0.4 to 1.2. During the temperature interval (160–200 °C) attributed to C-S-H decomposition, the mass losses in the samples were 0.6%, 0.9%, and 1.1%, respectively. During the temperature interval (200–250 °C) attributed to gaylussite decomposition, the mass losses in the samples were 0.8%, 1.0%, and 1.3%, respectively. It can be found that in the samples with C/S ratios of 0.4 and 0.75, the mass loss was significantly lower than that of 1.2. This could explain why no gaylussite was found in these two samples in the XRD study. Dehydration converted gaylussite to the double carbonate, Na_2_Ca(CO_3_)_2_, which generated the exothermic effects above 250 °C in the temperature intervals of 280–320 °C with mass losses of 0.8%, 0.9%, and 1.1%. Between 330 °C and 380 °C, another exothermic peak occurred due to crystal transformation from the low-temperature form of Na_2_Ca(CO_3_)_2_ to the high-temperature form with mass losses of 1.6%, 2.0%, and 3.8%, but the generally exothermic peaks in the temperature interval of 300–500 °C (including peaks at 400–500 °C) are due to the decomposition of BFA (combustion of soot and organic matter), and mass losses with increasing C/S ratio increased from 7% to 7.9% and 9.4% in the whole temperature interval. The decomposition of CaCO_3_ was observed during the endothermic effect (680–780 °C) with mass losses from 0.6% to 1.1%. The decomposition of Na_2_CO_3_ should be around 850–900 °C, but no clear endothermic peaks were identified, and the decomposition of sodium silicate occurs at temperatures above 1080 °C [108].

The total mass loss of the samples during the TG/DTA test varies significantly, with 17.0% for sample CS-0.40-40, 19.3% for sample CS-0.75-40, and 24.2% for sample CS-1.2-40. This result confirms that C-S-H and gaylussite were formed in the composition. Due to the synthesis of new products, the amount of CaCO_3_ is low. The overall results prove that the amount of synthetic products increases with increasing the C/S ratio in the composition.

### 3.10. Microstructure of BFA-Based AAM Pastes

**Figure 17:**
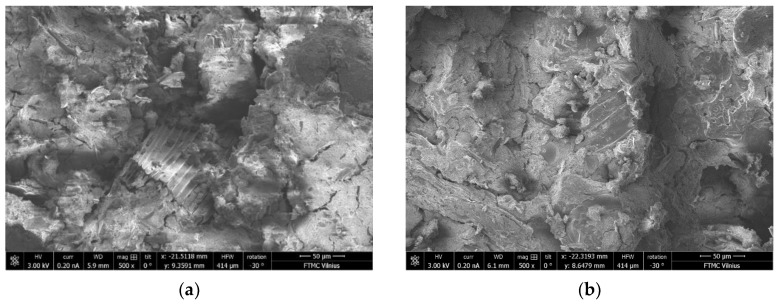
Microstructure of the samples cured at 20 °C: (**a**) CS-0.40-20; (**b**) CS-1.20-20 (magnification 500).

**Figure 18:**
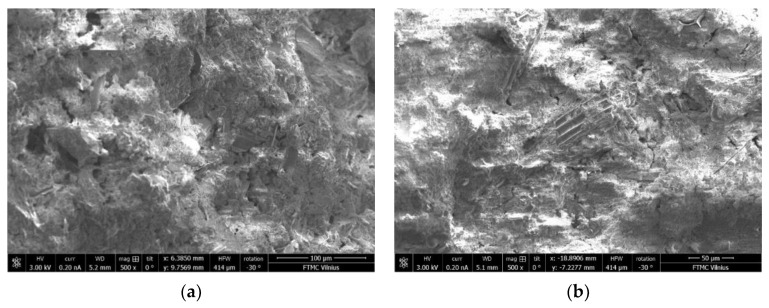
Microstructure of the samples cured at 40 °C: (**a**) CS-0.40-40; (**b**) CS-1.20-40 (magnification 500).

**Figure 19:**
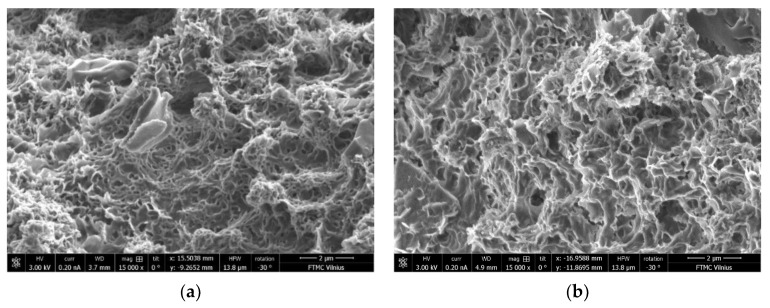
Microstructure of the samples cured at 20 °C: (**a**) CS-0.40-20; (**b**) CS-1.20-20 (magnification 15,000).

**Figure 20:**
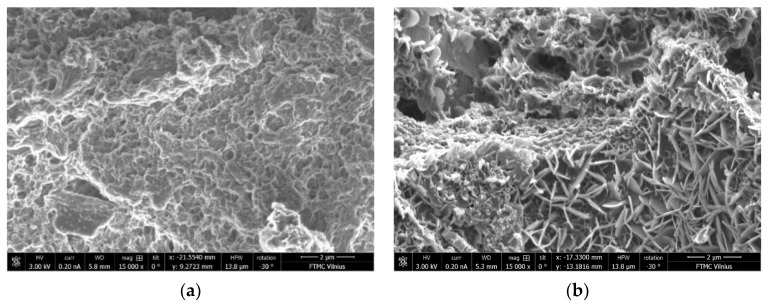
Microstructure of the samples cured at 40 °C: (**a**) CS-0.40-40; (**b**) CS-1.20-40 (magnification 15,000).

The differences in microstructure of the samples with C/S ratios of 0.40 and 1.20 cured at 40 °C and ambient temperature can be seen in the SEM images (Figure 17, Figure 18, Figure 19 and Figure 20). The sample with a C/S ratio of 0.40 cured at 20 °C (Figure 17a) has an uneven microstructure. The cracks and cavities are distributed around the unburned organic matter throughout the surface. The samples with a C/S ratio of 1.2 cured at 20 °C (Figure 17b) and 40 °C (Figure 18b) have fewer surface voids and denser structures. The larger visible voids and cracks could be linked with water evaporation from the gels during curing. The samples with smaller C/S ratios seem to have more unbound water in the structure, which significantly decreases the density and compressive strength of samples during evaporation.

Higher magnification shows that the sample cured at 20 °C (Figure 19) shows a highly porous gel structure when the C/S ratio is 0.40. When the C/S ratio is 1.20 in the sample, the gel structure has fewer visible pores. In contrast, the samples cured at 40 °C (Figure 20) exhibits a more compact microstructure with low void content, which could be responsible for their higher strength at 28 days. For samples cured at 40 °C, the gel structure of the sample with a C/S ratio of 0.40 is more even than that cured at 20 °C, and no cracks appeared. On the sample with a C/S ratio is 1.20 surface, the deposition of reaction products is evident. It shows that the reaction products have markedly changed, with calcium released from BFA and participating in activation reactions. The microstructure appeared to be denser than those pastes made with a C/S ratio of 0.4, and this observation supports its higher compressive strength. Overall, it can be underlined that the samples with good homogeneity and dense gel structure corresponded to compositions with higher compressive strength.

## 4. Conclusions

This study aims to investigate the possibility of utilizing unprocessed carbon-rich BFA in AAM. BFA-based AAM pastes with different Na_2_CO_3_/Na_2_SiO_3_ ratios at different curing temperatures (40 °C and 20 °C) were investigated in the study.

It has been established that with the increase in the amount of Na_2_CO_3_ in the solution, the electrical conductivity of the BFA-based AAM pastes increases and the initial and final setting times decrease. The AAM samples with the smallest C/S ratio are characterized by the lowest density and UPV value and the highest water absorption, whether they were cured at 40 °C or 20 °C. The SEM research confirms the density, water absorption, and UPV results, proving that with an increase in C/S ratio and curing temperature, the structure of the samples became denser and more homogenous, which corresponds with the higher compressive strength of the sample.

Regardless of the curing temperature, the compressive strength of AAM samples after curing for 28 days increased with the increase in the C/S ratio, thus indicating that the higher the alkali content, the better the mechanical properties. According to XRD analysis and TG/DTA tests, with the increase in the C/S ratio in the samples, the synthesis of gaylussite and C-S-H were observed. The formation of the mentioned minerals contributes to the compressive strength growth of the AAM paste with a higher C/S ratio. Overall, the compressive strength of the AAM samples cured at 40 °C was higher than that cured at 20 °C with the same composition. The higher the curing temperature, the faster the structure evolution during the BFA-based alkaline activation synthesis process.

The findings of this study contribute to the applicability of difficult-to-recycle waste materials such as BFA and the development of sustainable BFA-based AAM. The study results could also encourage the construction industry to use such materials to reduce the negative environmental impact of their storage.

## Figures and Tables

**Table 1:** Chemical compositions of BFA.

Element	Concentration (%)	Compound Formula	Concentration (%)
C	20.40	C	20.40
O	33.96	O	5.992
Na	0.192	Na_2_O	0.259
Mg	2.155	MgO	3.574
Al	1.390	Al_2_O_3_	2.627
Si	10.71	SiO_2_	22.91
P	1.182	P_2_O_5_	2.707
S	0.354	SO_3_	0.883
Cl	0.098	Cl	0.098
K	3.324	K_2_O	4.003
Ca	22.51	CaO	31.50
Ti	0.127	TiO_2_	0.212
Cr	0.011	Cr_2_O_3_	0.016
Mn	1.602	MnO	2.068
Fe	1.624	Fe_2_O_3_	2.322
Ni	0.010	NiO	0.013
Cu	0.013	CuO	0.017
Zn	0.080	ZnO	0.100
Rb	0.016	Rb_2_O	0.017
Sr	0.077	SrO	0.091
Y	0.015	Y_2_O_3_	0.019
Ba	0.154	BaO	0.172

**Table 2:** Composition of BFA-based AAM pastes (Group 1 and 2).

Group	Sample	Na_2_CO_3_ (wt% of BFA)	Na_2_SiO_3_ (wt% of BFA)	Na_2_CO_3_/Na_2_SiO_3_ Solute Ratio	Na_2_CO_3_/Na_2_SiO_3_ Solution Ratio	Curing Temperature (°C)
1	CS-0.40-40	3.00	7.50	0.40	0.67	40
	CS-0.60-40	3.75	6.25	0.60	1.00	40
	CS-0.75-40	4.17	5.56	0.75	1.25	40
	CS-0.90-40	4.50	5.00	0.90	1.50	40
	CS-1.20-40	5.00	4.15	1.20	2.00	40
2	CS-0.40-20	3.00	7.50	0.40	0.67	20
	CS-0.60-20	3.75	6.25	0.60	1.00	20
	CS-0.75-20	4.17	5.56	0.75	1.25	20
	CS-0.90-20	4.50	5.00	0.90	1.50	20
	CS-1.20-20	5.00	4.15	1.20	2.00	20

**Table 3:** EC and pH characteristics of Na_2_SiO_3_ solution and different Na_2_CO_3_ solutions.

Activator Solution	pH	EC, S/m
Na_2_SiO_3_	11.90	31.8
10% Na_2_CO_3_	11.36	71.4
20% Na_2_CO_3_	11.38	85.9
30% Na_2_CO_3_	11.38	79.2

## Data Availability

Not applicable.
